# PPAR/RXR Regulation of Fatty Acid Metabolism and Fatty Acid *ω*-Hydroxylase (CYP4) Isozymes: Implications for Prevention of Lipotoxicity in Fatty Liver Disease

**DOI:** 10.1155/2009/952734

**Published:** 2010-03-16

**Authors:** James P. Hardwick, Douglas Osei-Hyiaman, Homer Wiland, Mohamed A. Abdelmegeed, Byoung-Joon Song

**Affiliations:** ^1^Biochemistry and Molecular Pathology, Department of Integrative Medical Sciences, Northeastern OH Universities College of Medicine and Pharmacy (NEOUCOM/NEOUCOP), 4209 state Route 44, Rootstown, OH 44272, USA; ^2^Laboratory of Physiologic Studies, National Institute on Alcohol Abuse and Alcoholism, NIH, 9000 Rockville Pike, Bethesda, MD 20892-9410, USA; ^3^CardioMetabolic Disease Research Group, Department of Molecular and Cellular Biology, Kobe Pharma Research Institute, Nippon Boehringer Ingelheim Co., Ltd, 6-7-5 Minatojima-Minaminachi, Chuo-Ku, Kobe 650-0047, Japan; ^4^Laboratory of Membrane Biochemistry and Biophysics, National Institute on Alcohol Abuse and Alcoholism, NIH, 9000 Rockville Pike, Bethesda, MD 20892-9410, USA

## Abstract

Fatty liver disease is a common lipid metabolism disorder influenced by the combination of individual genetic makeup, drug exposure, and life-style choices that are frequently associated with metabolic syndrome, which encompasses obesity, dyslipidemia, hypertension, hypertriglyceridemia, and insulin resistant diabetes. Common to obesity related dyslipidemia is the excessive storage of hepatic fatty acids (steatosis), due to a decrease in mitochondria *β*-oxidation with an increase in both peroxisomal *β*-oxidation, and microsomal *ω*-oxidation of fatty acids through peroxisome proliferator activated receptors (PPARs). How steatosis increases PPAR*α* activated gene expression of fatty acid transport proteins, peroxisomal and mitochondrial fatty acid *β*-oxidation and *ω*-oxidation of fatty acids genes regardless of whether dietary fatty acids are polyunsaturated (PUFA), monounsaturated (MUFA), or saturated (SFA) may be determined by the interplay of PPARs and HNF4*α* with the fatty acid transport proteins L-FABP and ACBP. In hepatic steatosis and steatohepatitis, the *ω*-oxidation cytochrome P450 *CYP4A* gene expression is increased even with reduced hepatic levels of PPAR*α*. Although numerous studies have suggested the role ethanol-inducible *CYP2E1* in contributing to increased oxidative stress, *Cyp2e1*-null mice still develop steatohepatitis with a dramatic increase in *CYP4A* gene expression. This strongly implies that *CYP4A* fatty acid *ω*-hydroxylase P450s may play an important role in the development of steatohepatitis. In this review and tutorial, we briefly describe how fatty acids are partitioned by fatty acid transport proteins to either anabolic or catabolic pathways regulated by PPARs, and we explore how medium-chain fatty acid (MCFA) *CYP4A* and long-chain fatty acid (LCFA) *CYP4F* *ω*-hydroxylase genes are regulated in fatty liver. We finally propose a hypothesis that increased *CYP4A* expression with a decrease in *CYP4F* genes may promote the progression of steatosis to steatohepatitis.

## 1. Introduction

Disorders of lipid metabolism are closely dependent on genetic factors, exposure to drugs, and many common life-style choices (e.g., diets and alcohol) that often lead to metabolic syndrome in which patients exhibit obesity, dyslipidemia, hypertension, hypertriglyceridemia, and insulin resistance diabetes [1, 2]. Common to obesity-related dyslipidemia and hypertriglyceridemia is the excessive storage of fatty acids in the liver (steatosis) frequently referred as nonalcoholic fatty liver disease (NAFLD). Increased hepatic fatty acids can cause lipotoxicity and initiate fatty liver inflammation (steatohepatitis) commonly referred as nonalcoholic steatohepatitis (NASH) [3]. Excessive fatty acids in the liver dramatically alter lipid metabolism by decreasing mitochondrial *β*-oxidation, while increasing peroxisomal *β*-oxidation and microsomal *ω*-oxidation of fatty acids resulting in lipotoxicity [4, 5]. During hepatic steatosis, members of *CYP4 *family of fatty acid *ω*-hydroxylase are induced even with the downregulation of PPAR*α*, which regulates *CYP4A *gene expression. Numerous reports have indicated that the ethanol-inducible CYP2E1 is induced in both hepatic steatosis and steatohepatitis [6, 7] even though *Cyp2e1*-null mice develop steatohepatitis with a markedly increased *CYP4A* gene expression, suggesting that *CYP4A* P450 may also play an important role in the progression of NAFLD to NASH. Since elevated levels of long chain fatty acids (LCFAs) and LCFA coenzyme A esters (LCFA-CoAs) are observed in hepatic steatosis and several metabolic disorders, including obesity, diabetes and hyperlipidemia, it is of necessity that we understand the mechanism that regulates fatty acid (FA) transport and partitioning of free fatty acids (FFA) and fatty acid-CoA in the initiation of fatty liver lipotoxicity in the progression of NAFLD to NASH. 

Intracellular fatty acids (FAs) and their metabolites coordinate physiological processes by several transcriptional factors controlling energy metabolism. For instance, several transcriptional factors include: peroxisome proliferator activated receptors (PPAR*α*, PPAR*δ*, PPAR*γ*), sterol regulatory binding proteins (SREBP-1 and SREBP-2), liver X receptor (LXR*α*), and carbohydrate response element binding protein (ChREBP), all of which are activated or repressed by different fatty acids [8]. Both polyunsaturated fatty acids (PUFAs) eicosapentaenoic acid (EPA, C20:5n-3) and docosahexaenoic acid (DHA, C22:6n-3) and LCFA-CoA bind and activate PPAR*α* to increase fatty acid oxidation, gluconeogenesis, and ketogenesis [9], while PUFAs suppress activation of SREBP-1c, ChREBP, and LXR*α* through diverse mechanisms [10]. In contrast, saturated fatty acids activate HNF4*α* through binding to acyl-CoA binding protein (ACBP) containing LCFA while SREBP-1c activation by LCFA occurs by recruiting SREBP-1c and PPAR*γ* coactivator-1*β* (PGC-1*β*). In addition, intracellular fatty acids produced from triglyceride hydrolysis or de novo lipogenesis (DNL) can regulate gene expression, suggesting that not only the type of fatty acids (saturated versus unsaturated), sources (intracellular versus exogenous), fatty acid association with different fatty acid transport proteins (L-FABP and ACBP), and type of fatty acid metabolites (FATP/ACSVL) have selective effects in regulating genes involved in oxidation, synthesis, and storage of fatty acids [10, 11]. Perturbations in these pathways by nutritions, drugs, alcohol, or genetic factors lead to fatty acid disorders often associated with dyslipidemia, obesity, and diabetes [12–14]. 

## 2. Causes of Hepatic Steatosis in NAFLD

Convincing evidence has shown that fatty liver is closely associated with insulin resistance and metabolic syndrome. Although, life style choices of a high carbohydrate or a high fat diet and excessive alcohol consumption are specific causes of hepatic steatosis and NAFLD, nutritional factors (e.g., malnutrition and rapid weight loss) [15], drug exposure (e.g., glucocorticoids, methotrexate, Amiodarone, Tamoxifen, HIV protease inhibitors, etc.) [12, 16], specific diseases (e.g., inflammatory bowel disease, primary biliary cirrhosis, Cushing's syndrome, Hematochromatosis, etc.), and genetic factors [17] are also relevant causes of fatty liver diseases [16, 18–22]. 

The major sources of fatty acids that contribute to hepatic steatosis include fat stored in adipose tissues and released during fasting by lipolysis to increase plasma levels of nonesterified fatty acid (NEFA), which provides the majority of fatty acids secreted by liver as VLDL. During adipose insulin resistance, the increased release of fatty acids leads to elevated accumulation of TAG in fatty liver. A second source of increased plasma NEFA is through liver de novo lipogenesis (DNL) while dietary fatty acid, from intestinal-derived chylomicron remnants, also increases the plasma pool of NEFAs. In patients with NAFLD, 59% of TAG fatty acid is derived from adipose lipolysis while 26% is from DNL and 15% from the dietary NEFA pool [21]. The elevated DNL in patients with NAFLD compared to patients without NAFLD does not change after a meal. In contrast, in normal control individuals it increases from 5% to 28% 4 hours after a meal [22]. In NAFLD, the NEFA pool contributes equally to the liver TAG and VLDL TAG even though NAFLD patients have a reduced ability to increase VLDL production during fasting, suggesting that NAFLD patients have a limited capacity to adapt to metabolic changes that occur during cycles of fasting and feeding [23]. 

Thus, plasma NEFAs from adipose stores or dietary sources provide most of the hepatic lipid in NAFLD. Therefore, the regulatory mechanism of fatty acid uptake by hepatocytes and cellular distribution of esterified and nonesterified fatty acids have an important role in the initiation of hepatic steatosis in NAFLD. There are five mechanisms responsible for fatty acid uptake that initiates of fatty liver. Facilitated transport is the primary mechanism for the uptake of free fatty acids (FFAs) into hepatocytes under normal plasma NEFA levels (225–700 uM). However, higher plasma NEFA concentrations may overload this saturable system, resulting in the activation of a passive nonsaturable pathway to reduce plasma NEFA levels, unfortunately with the consequence of hepatic steatosis [24]. The saturable systems for plasma membrane fatty acid transport are caveolins, fatty acid transport proteins (FATPs), fatty acid translocase (FAT/CD36), and fatty acid binding proteins (FABPs). Caveolin-1 of lipid rafts is localized within plasma membrane invaginations that have a critical role in cell signaling, protein trafficking, and uptake of fatty acids; caveolin-1 deficient mice are resistant to diet induced obesity [25]. FATPs are a family of six integral membrane proteins with an extracellular/luminal N-terminal and C-terminal domain with *fatty acyl-CoA synthetase* activity and therefore FATP proteins have the ability to trap FA intracellularly [26] (Table 1). FATP2 (ACSVL1), FATP5 (ASCLV6), and FATP4 (ACSVL5) are expressed in the liver and are regulated by PPAR*α* and PPAR*γ* [27]. Both FATP2 and FATP4 have a strong substrate preference in transport and activation of C16:0 to C24:0 straight chain and branched-chain fatty acids while FATP5 (ACSVL6) preferentially transports and activates bile acids [26]. FATP5 knockout mice show a 50% decrease in hepatocyte fatty acid uptake with reduced caloric uptake, and improved whole body glucose homeostasis. Consequently, these mice were protected from high fat induced hepatic steatosis [28]. FATP5 also exhibits *bile acid CoA synthetase* activity and therefore *Fatp5*-null mice display a dramatic increase in unconjugated bile acids. As expected *Fatp5*-null mice are resistant to obesity and hepatic accumulation of TAG with improved insulin sensitivity [28, 29]. In adenovirus FATP4 infected rat hepatocytes, there was a 30% increase in fatty acid uptake and 2-fold increase in acyl-CoA activity with a 42% increase in TAG synthesis, indicating that FATP4 partitions fatty acids towards TAG synthesis and storage [30–32]. In human hepatocytes, FATP4 knockdown decreased C_18:1_ incorporation into phospholipids and VLDL synthesis, suggesting an anabolic role of FATP4 in energy metabolism [33]. In contrast, overexpression of FATP2 in primary hepatocytes results in C_18:1_ channeling toward diacylglycerol and phospholipid synthesis with shift away from cholesterol esterification [33]. FATP2 is regulated by PPAR*α* agonists as well as high carbohydrate and high fat diets which increase FATP2 expression 8-fold in rat liver. However, surprisingly the PPAR*γ* ligand BRL-49953 induces FATP2 expression in adipose tissue, which is similar with insulin induced FATP2 expression [34]. It is therefore not surprising that FATP2 is induced in obese Zucker (fa/fa) rats and that both FATP2 and FATP4 mRNA are increased in hepatocytes by carbohydrates feeding and by insulin through SREBP-1c [35]. FAT/CD36 is expressed in a broad range of tissues and cell types. It promotes fatty acid release from albumin and insertion into the plasma membrane by facilitated diffusion with the assistance of L-FABP. Although expression of CD36/FAT is low in hepatocytes, this transport protein is unique since it mediates the uptake of VLCL and oxidized LDL. *Cd36*-null mice show a higher level of plasma NEFA, higher liver TAG, and severe hepatic insulin resistance [36]. The induction of CD36/FAT in mice fed a high fat diet results in fatty liver [37], and patients with increased expression of this protein have a higher level of hepatic fatty acids and display NAFLD [38]. CD36/FAT expression is controlled by the pregnane X receptor (PXR), PPAR*γ*, and the LXR*α* [37]. 

FABP and ACBP bind a diverse array of fatty acids including eicosanoids [39] and facilitate intracellular transport of FA and FA-CoAs from the cytosol to different organelles including the nucleus. Unlike FATP, this protein does not exhibit *acyl-CoA synthetase* activity (Table 1). There are nine different FABPs each of which shows distinct tissue specific distribution with L-FABP being prominently expressed in the liver. *L-Fabp*-null mice show a 2-fold increase in hepatic TAG compared to a 10-fold increase in wild type mice after a 48-hour fasting, which was due to decreased TAG secretion and reduced fatty acid oxidation [40]. *L-Fabp*-null mice fed a high fat western diet are resistant to diet induced obesity and show a similar increase in TAG secretion as the wild type mice [41], suggesting either that other fatty acid transport proteins compensate for L-FABP or increased plasma NEFAs initiate passive diffusion. A high fat diet potently increases the expression of L-FABP [42] and microsomal triglyceride transfer protein (MTP) via PPAR*α*, thereby leading to efficient delivery of FA for VLDL assembly and secretion [43]. In contrast, the repression of these enzymes would reduce VLDL secretion without causing TAG accumulation in the liver [44]. ACBP, responsible for transporting acyl-CoA esters intracellularly, is downregulated during fasting but induced by insulin through SREBP-1c, and fibrates through PPAR*α* [45]. Thus ACBP is a dual PPAR*α* and SREBP-1c target gene where fasting reduces SREBP-1c expression and increases PPAR*α* in hepatocytes, thus reflecting a dual role of ABCP in lipogenesis and lipid oxidation. 

The apparently similar mechanism that regulates FATP2, FATP4, ACBP, and L-FABP by PPAR*α* during fasting induced fatty acid influx with the paradoxical upregulation by insulin through SREBP-1c identifies an ideal mechanism to prevent lipotoxicity [46]. Cellular acyl-CoA levels correlate with hepatic insulin resistance and have been suggested to mediate lipotoxicity. Because the normal acyl-CoA levels in cytosol are between 1 and 20 uM, and ACBP and FABP comprise up to 5% of the total hepatic cytosol proteins [34], it is likely that most acyl-CoAs are bound to ACBP and/or FABP and thus FFAs may be the major culprit in causing lipotoxicity. Since LCFA-CoA production is controlled by FATPs, which channel acyl-CoAs to the synthesis of phospholipids, cholesterol esters, and triglycerides or oxidation by the mitochondrial and peroxisomal *β*-oxidation pathways or microsomal *ω*-oxidation, it is highly likely that elevated NEFAs in serum during fasting and hepatic steatosis significantly increase the intracellular pool of unesterified fatty acids in hepatocytes. The increased synthesis of TAG observed in hepatic steatosis may be a compensatory mechanism to reduce intracellular FFAs and lipotoxicity. Therefore, the upregulated FATP2, FATP4 and FATP5 expression in obese rats increases TAG synthesis and storage, while increased FATP expression promotes the cellular importation of FFAs [33, 47], leading to FFA overload and hepatic steatosis. However, the knockdown of FATP3 (ACSLV3) in primary rat hepatocytes revealed a significant reduction in the expression of several lipogenic transcription factors, PPAR*γ*, ChREBP, SREBP-1c, and LXR*α* as well as their target genes [46, 48]. 

Since the knockdown (or knockout) of different FATP isoforms reduces hepatic steatosis and in some cases increases insulin sensitivity and glucose handling, it is imperative that we understand the mechanism through which various FATP isoforms interact with different metabolic enzymes and/or intracellular transport proteins (L-FABP and ACBP) to regulate lipogenesis, TG synthesis and storage, and fatty acid oxidation pathways. These mechanistic studies will provide valuable insights into how hepatic steatosis causes lipotoxicity and the progression of steatosis to steatohepatitis. 

## 3. Role of PPAR in the Regulation of Fatty Acid Metabolic Fate

The overexpression of various fatty acid transport proteins in different cell lines, and their ability to channel FA to different metabolic fates within the cell [49] may be determined by the energy demands of specific metabolic pathways. Indeed, FATP proteins are not only associated with the plasma membrane but are also present in specific organelles (Table 1). FATP5 is intimately associated with the mitochondria while FATP2 (ACSVL1) is localized in peroxisomes. CD36/FAT is closely correlated with the degree of mitochondrial fatty acid oxidation, while L-FABP, also known as the mitochondrial aspartate aminotransferase (mAST), may have distinct functions at different subcellular sites. Unlike FATP which activates FA to FA-CoA, FABP proteins transport fatty acids to different intracellular compartments. The association of FATP with different subcellular organelles may also function in preventing lipotoxicity through increased metabolism. This mechanism may be important in hepatic fatty acid partitioning to different metabolic pathways during hepatic steatosis. Recent studies have shown that L-FABP also functions to shuttle lipids to the nucleus to allow them to directly interact with PPAR*α* [49], suggesting an important role of L-FABP in controlling the metabolism of LCFAs. The ability of fatty acids to regulate metabolic pathways through activation of nuclear receptors has long been known; however the observation that L-FABP assists in supplying lipid ligands for nuclear receptors suggests an important feedback regulatory mechanism of L-FABP and FAs in controlling lipid metabolism. 

**Figure 1:**
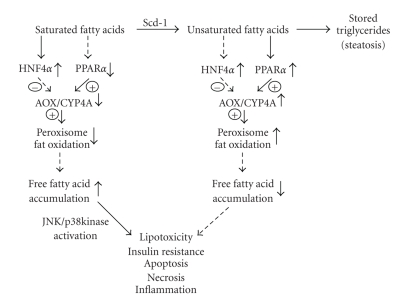
Schematic diagram of the role of saturated fatty acids in causing nonalcoholic fatty liver diseases and lipotoxicity. The positive signs with solid lines represent activation and/or upregulation of the downstream targets while the negative signs with broken lines indicate the opposite effects. Abbreviations used are HNF4*α*, hepatocyte nuclear factor 4; PPAR*α*, peroxisome proliferator activator receptor *α*; AOX, acyl-CoA oxidase; CYP4A, cytochrome P450 4A; Scd-1, stearoyl-CoA desaturase.

The ability of FATPs to target fatty acids to specific cellular organelles for either fatty acid oxidation or synthesis and their ability to direct fatty acid ligands to activate selective nuclear receptors represent an efficient mechanism to control both metabolic pathways at the transcriptional and substrate levels. The activation of hepatocyte nuclear factor 4*α* (HNF4*α*) and PPAR*α* that bind similar direct repeat DNA elements (DR1) by different fatty acids suggests an efficient mechanism to control globally fatty acid metabolism [50, 51] (Figure 1). It has been shown that saturated LCFA and VLCFA are extremely poor or nonactivators of PPAR*α*. Therefore it was uncertain how increased hepatic fatty acids during fasting would increase PPAR*α* target genes involved in fatty acid *β*-oxidation, VLDL production, and ketogenesis. This was resolved by demonstration that LCFA-CoA and VLCF-CoA are high affinity PPAR*α* ligands. PPAR*α* has a high affinity for polyunsaturated fatty acids (PUFAs) and LCFA-CoA, while these saturated LCFAs or VLCFAs are nonselective in the activation of PPAR*α*, PPAR*δ*, and PPAR*γ*. In vivo, the importance of acyl-CoA in the activation of PPAR*α* target genes was apparent when it was found that CoA esters of peroxisome proliferator chemicals or drugs were activating ligands for PPAR*α* mediated expression of peroxisomal fatty acid *β*-oxidation genes, Acyl-CoA oxidase (AOX1), bifunctional protein and thioesterase [52–54]. It was also found that peroxisome proliferator-CoA esters (PP-CoAs) are potent inhibitors of HNF4*α* activation. The importance of VLCFA-CoAs in activation of PPAR*α* was shown in the in vivo animal model of *Aox1*-null mice, which show elevated plasma levels of VLCFAs and increased hepatic levels of VLCFA-CoAs. The inability of peroxisomal *β*-oxidation system to metabolize VLCFA-CoAs results in hyperactivation of PPAR*α* and increased expression of target genes [55]. In contrast, HNF4*α* has a high affinity for saturated LCFAs, and VLCFAs, but not PUFA-CoAs or LCFA-CoAs, suggesting that fatty acid CoA, chain length, and degree of unsaturation determine whether HNF4*α* or PPAR*α* will be activated and therefore which metabolic pathway controlled by these nuclear receptors [10, 56]. Thus, the elevated uptake and transport of LCFA-CoAs or PUFAs to the nucleus by L-FABP activate PPAR*α* and increase fatty acid oxidation. In contrast, ACBP binds saturated LCFAs, which preferentially bind and activate HNF4*α*. Therefore, PPAR*α*/L-FABP and HNF4*α*/ACBP would mediate a differential association with coactivators and corepressors to their respective target genes to control fatty metabolism. This suggests that binding of saturated LCFAs to ACBP and its association with HNF4*α* would increase HNF4*α* transcription activity and inhibit PPAR*α*, while transactivation of PPAR*α* with PUFAs or LCFA-CoAs would decrease HNF4*α* activation [10]. Thus, in hepatic steatosis, the ratio of saturated to unsaturated fatty acids with increased expression of selective fatty acid transport proteins (FATP/L-FABP/ACBP) may mediate metabolic defects accounting for hepatic steatosis. Consequently, LCFA activation of HNF4*α* with ACBP would increase plasma levels of lipid rich lipoproteins (VLDL, LDL, and HDL) and their constituent apolipoproteins (AI, AII, B, and CIII). In contrast, dietary PUFAs associated with L-FABP would activate PPAR*α*, resulting in decreased transcription of these apolipoproteins and lipoproteins with increased fatty acid oxidation [52, 53, 57]. It will be of significance to determine if selective fatty acids and fatty acid transport proteins regulate the expression of PPAR*γ*, LXR*α*, and SREBP-1c in hepatic steatosis. Recently, the suppression of FATP3 (ACSVL3) was shown to significantly decrease the expression of lipogenic transcription factors, PPAR*γ*, ChREBP, LXR*α*, and SREBP1c and their respective target genes, resulting in decreased de novo lipogenesis (DNL) [46]. In hepatic steatosis, many of the fatty acid transporter proteins are upregulated, yet it is uncertain how this common event of increased hepatic fatty acid levels seen in fasting leads to increased fatty liver and lipotoxicity in the progression of NAFLD to NASH. 

## 4. Fatty Acid-Induced Lipotoxicity

Elevated intracellular fatty acids in hepatocytes lead to lipotoxicity characterized by increased oxidative-stress and lipid peroxidation, thus promoting the progression of simple hepatic steatosis to steatohepatitis. Normal cellular fatty acid homeostasis represents a balance between fatty acid uptake, utilization, and export from the liver, which is controlled by an elaborate transcriptional network that is finely tuned to meet the energy needs of cells to prevent the accumulation of fatty acids and toxic intermediates. However, when this system is overwhelmed by excessive free fatty acids (FFAs), hepatic steatosis commonly referred as NAFLD can progress to steatohepatitis often referred as NASH. NASH is characterized by cellular damage, inflammation, and varying degrees of fibrosis [58]. Untreated NASH can progress to liver fibrosis, contributing to hepatorenal portal hypertension, liver cirrhosis, hepatic encephalopathy, and liver failure or progress to hepatocellular carcinoma [59, 60]. In the liver, FFAs are considered the causative agents for hepatic steatosis and also for obesity, and diabetes. Therefore, elucidating the mechanism by which excessive hepatic FFAs induce hepatic insulin resistance, hepatic gluconeogenesis, and fatty acid disposal or intracellular partitioning is critical for understanding the progression of steatosis to steatohepatitis. Serum FFAs levels are increased in obese individuals in both the fed and fasting states and have been shown to play a critical role in the progression of obesity to Type II diabetes [61]. FFAs in the liver desensitize insulin signaling by dampening suppression of hepatic gluconeogenesis through activation of p38 mitogen-activated kinase [62]. FFAs increase the transcription of phosphoenolpyruvate carboxykinase (PEPCK), glucose 6-phosphatase (G6Pase), peroxisome proliferator-activated receptor coactivator *α* (PGC-1*α*), and cAMP-responsive element binding protein (CREBP). Activation of p38 kinase by FFAs is mediated by upstream activation of protein kinase C*δ* (PKC*δ*), which might be activated by ACSLV-directed FFA diversion to diacylglycerol (DAG), which is known to activate PKC*δ* in the presence of calcium. 

Hepatic insulin resistance represents a paradox in hepatic steatosis since insulin receptor activation of insulin receptor substrate-1 (IRS-1) is active and accounts for increased DNL, yet insulin does not inhibit insulin receptor substrate-2, which controls liver gluconeogenesis [63]. Both IRS-1 and IRS-2 inhibit the transcription factor FOXO-mediated gene transcription of PEPCK, G6Pase, and PGC-1*α* [64]. Increased activation of IRS-1 during hyperinsulinemia increases expression of SREBP-1c, which normally blocks IRS-2 expression and CREBP critical for hepatic gluconeogenesis. Excessive FFAs induce hepatic insulin resistance by activation of c-Jun N-terminal protein kinase (JNK), which is known to be activated by oxidative stress and cytokines, and is abnormally elevated in the diabetics [7, 65]. Thus, inhibition of JNK dramatically improves insulin resistance and markedly decreases blood glucose levels. JNK adenovirus overexpression increases serine phosphorylation of IRS-1 with decreased IRS-1 tyrosine phosphorylation and increased SREBP-1c activation, resulting in increased fat synthesis and storage. JNK-mediated decreased activation of IRS-1 also results in reduced suppression of FOXO1, leading to increased hepatic gluconeogenesis. However, a recent study reported that specific ablation of JNK1 in hepatocytes exhibits glucose intolerance, insulin resistance, and hepatic steatosis [66]. This study suggests that JNK1 has opposing actions in liver and adipose tissue to promote and prevent hepatic steatosis. Liver specific *Jnk1*-null mice display increased gluconeogenesis and lipogenesis and, therefore, have the paradox of selective insulin resistance that is a central characteristic of type 2 diabetes. JNK activation has also been implicated in FFA-induced hepatocyte lipoapoptosis by activation of proapoptotic BCL-2 proteins Bim and Bax, triggering the mitochondrial apoptotic pathway [4, 67]. Furthermore, IRS-2 signaling was repressed in hepatic insulin resistance by short-term feeding of FFAs, which caused a 3-fold increase in triglycerides and acyl-CoAs. Not only was tyrosine phosphorylation of IRS-2 decreased, but also IRS-1 activation was suppressed in mice fed a high fat diet. Consequently, the reduced tyrosine phosphorylation of IRS-1 and IRS-2 in rat hepatoma cells exposed to palmitic acid can be reversed by inhibition of acyl-CoA synthesis of palmitoyl-CoA, suggesting an important role of FATP (ACSLV) proteins in insulin signaling [50]. 

Although liver insulin resistance and hepatocyte apoptosis are induced by excessive FFAs, the question of which types of fatty acids promote each process has not been explored in mouse models of hepatic steatosis. Recent data suggested that partitioning of saturated and unsaturated fatty acids through *stearoyl CoA desaturase* (SCD-1) determines the degree of fatty acid induced liver injury [68]. SCD-1 knockout mice are resistant to hepatic steatosis and hepatic insulin resistance [69, 70]. SCD-1 regulates partitioning of saturated fatty acids (SFAs) between MUFAs present in simple hepatic steatosis and SFAs present during hepatic steatohepatitis and fibrotic livers. In an elegant study, Feldstein and colleagues clearly showed that MUFA leads to hepatic steatosis without hepatocyte injury while SFA significantly decreases hepatocyte cell viability through caspase activation and apoptosis [71]. Furthermore, inhibition of SCD-1 sensitized hepatocytes to SFA-induced apoptosis, and mice fed with a high-fat diet showed increased SCD-1 expression and hepatic accumulation of MUFA. In contrast, mice fed with a methionine-choline deficient (MCD) diet that induces steatohepatitis had increased hepatic levels of SFAs. *Scd1*-null mice fed with the MCD diet showed decreased hepatic steatosis, but increased apoptosis and liver fibrosis, which could be prevented by feeding MUFA. Therefore, although *Scd1*-null mice are resistant to hepatic steatosis, they are more susceptible to liver injury while increased SCD-1 expression during NAFLD reflects a compensatory beneficial effect on increasing MUFA synthesis and storage of excessive FFAs as triglycerides. Therefore, a decreased fatty acid desaturation index (MUFA/SFA) may be one important trigger in the progression of NAFLD to NASH. In addition, a recent report revealed that the SFA C12:0 is a potent Toll-like receptor four (TLR4) activator [72]. Toll-like receptors are pattern-recognition receptors that induce the innate and adaptive immune responses in mammals, and therefore their activation by SFAs may initiate steatohepatitis. It is interesting that PUFAs inhibit TLR4 dimerization, which promotes the production of NADPH oxidase-dependent ROS, which is believed to be the second hit in the progression of steatosis to steatohepatitis. 

## 5. Mitochondrial Fatty Acid Oxidation in Hepatic Steatosis

In hepatic steatosis it is believed that mitochondrial dysfunction and decreased fatty acid *β*-oxidation are precipitating causes for increased intracellular FFA accumulation and hepatic insulin resistance. It is of interest that mitochondrial *β*-oxidation is active in patients with NAFLD, and therefore the question of whether excessive influx of FFAs and overproduction of ROS promote mitochondrial injury or simply represent the consequences of abnormal fatty acid metabolism needs further investigation. Mitochondrial *β*-oxidation is primarily responsible for the oxidation of short chain (SCFA < C_8_), medium chain (MCFA, C_8_–C_12_), and long chain (LCFA, C_12_–C_18_) fatty acids to acetyl-CoA, which can be condensed into ketone bodies, oxidized to CO_2_ and water, or serve as the building block for lipid synthesis. Mitochondrial *β*-oxiation is regulated by carnitine palmitoyltransferase (CPT1), carnitine concentration, and malonyl-CoA produced by acetyl CoA carboxylase (ACC2) from cytosolic acetyl-CoA. ACC2 gene expression is induced by SREBP-1a, and therefore *Srebp-1a*-null mice have lower hepatic levels of triglycerides and higher serum levels of ketone bodies. SREBP-1a levels are increased in patients with NAFLD and may provide an important mechanism to prevent efficient *β*-oxidation of fatty acids by increased ACC2-mediated production of malonyl-CoA and inhibition of CPT1. ACC2 knockout mice have a greater fatty acid oxidation rate, with reduced fat mass and enhanced insulin sensitivity [73]. The initial step in mitochondrial *β*-oxidation is the dehydrogenation of acyl-CoA esters by a family of 4 chain length specific straight chain acyl-CoA dehydrogenases. Mice with disrupted MCFA or LCFA acyl-CoA dehydrogenase develop micro- and macrovascular hepatic steatosis [74]. In human with defects in mitochondria trifunctional protein complex (MTPr), consisting of 2-enoyl CoA hydratase, 3-hydroxyacyl-CoA dehydrogenase, and 3-ketoacyl CoA thiolase activities, hepatic steatosis develops quickly after birth, leading to premature death [75]. 

How lipotoxicity causes oxidative stress and mitochondria dysfunction is an active area of research with regards to palmitic acid toxicity. It is known that C_16:0_ is a poor substrate for diacylglycerol acyltransferase, the last step in TAG synthesis; therefore, C_16:0_ is used instead in the synthesis of ceramide, which can induce NADPH oxidase and disrupt mitochondrial respiration either by inducing mitochondrial release of cytochrome c or by disruption of the respiratory chain complex III [76]. However, DAG through activation of protein kinase C-dependent pathways is also able to activate NADPH oxidase and initiate FFA-induced apoptosis. However, it is uncertain whether oxidative stress from NADPH oxidase, cytochrome P450, or the mitochondria is the main contributing factor in the progression of steatosis to steatohepatitis. Treating rat H4IIEC3 hepatoma cells with either C_18:1_ MUFA or C_16:0_ SFA revealed that palmitic acid, but not oleate, inhibited IRS-2 tyrosine phosphorylation and serine phosphorylation of AKT, through JNK activated by mitochondria-derived ROS [77]. Thus, mitochondria-derived ROS induced by palmitic acid may be a major contributor to JNK activation and hepatic insulin resistance. However, whether a similar mechanism occurs in animal fed with a high-fat saturated fatty acid diet will have to be determined. Indeed, mitochondria isolated from mice fed a high fat diet show depressed state-3 respiration, decreased uncoupled respiration, and decreased cytochrome c oxidase activity with no change in complex-I-mediated ROS production [78]. A reduced membrane potential of mitochondria from mice fed with a high fat diet suggests that mitochondria may not be the major source of ROS in hepatic steatosis, which is contradictory to the results observed with cultured hepatoma cells [77]. Unlike animal studies, in humans with NAFLD, whole body lipid oxidation is increased because of peripheral insulin resistance, suggesting that impairment in hepatic fatty acid oxidation does not seem to contribute to hepatic steatosis in humans [79], although these data need further confirmation. Therefore, the role of mitochondrial fatty acid oxidation in the liver seems controversial, with fatty acid oxidation viewed as a protective mechanism of disposal of potentially toxic FFAs, although increased oxidation of fatty acids can generate ROS, which may initiate steatohepatitis. Fatty acids impair mitochondrial function in human primary hepatocytes through disruption of the respiratory chain activity. This may increase ROS production in NAFLD patients, with increased fatty acid *β*-oxidation, resulting in uncoupling of the electron transport to oxidative phosphorylation [3, 80]. In addition to functional abnormalities in both experimental models of hepatic steatosis and in human patients with NAFLD/NASH, mitochondrial morphological changes have been observed in steatohepatitis but not steatosis. These morphological changes are believed to be due to oxidative stress-induced phospholipid phase transition that is seen as crystalline mitochondria inclusions [81]. Thus, the upstream events that lead to mitochondrial dysfunction and whether mitochondria are the source of ROS in NAFLD/NASH are largely unknown. Indeed moderate mitochondrial dysfunction in the respiratory chains may be of benefit in protecting against obesity and diabetes, by inducing the expression of mitochondrial uncoupling proteins (UCPs) and dissipation of mitochondria membrane potential. 

Mitochondrial uncoupling proteins (UCP2 and UCP3) are believed to function to increase mitochondrial conductance when activated by ROS. UCP gene expression is increased in hepatic steatosis and evidence supports a role for UCP3 in fatty acid metabolism by exporting fatty acid anions, and thus maintaining mitochondria CoA levels [82]. UCP3 was found to be necessary for fasting-induced increase in fatty acid oxidation rate and capacity through mitigation of mitochondrial oxidative stress [30]. When there is an excess of FFA, either through passive transport of shorter chain fatty acids from peroxisomes or ACSVL activation and CPT1 transport that exceed the mitochondrial fatty acid oxidation potential, there is a greater risk of mitochondrial lipid peroxidation, which can be prevented by UCP3-mediated efflux of fatty acids [83, 84]. Thus, the increase in ACSLV and UCP3 proteins in hepatic steatosis is an adaptive protective mechanism to prevent mitochondrial lipotoxicity [85].

## 6. Peroxisomal Fatty Acid Oxidation in Hepatic Steatosis

The peroxisomal *β*-oxidation system metabolizes very long-chain saturated and unsaturated fatty acids (VLCFA, >C_18_), prostaglandins, and leukotrienes. Peroxisomes also metabolize branched-chain fatty acids, dicarboxylic acid produced by the microsomal *ω*-oxidation, and C_27_ bile acid intermediates. Unlike mitochondrial *β*-oxidation, peroxisomal *β*-oxidation does not completely oxidize FAs to CO_2_ and water but rather chain shortens FAs by 2 or 3 cycles of the *β*-oxidation. The peroxisomal *β*-oxidation consists of a *P* *P* *A* *R* *α* inducible system that metabolizes straight-chain saturated fatty acids and a noninducible pathway that metabolizes branched-chain fatty acids and bile acid intermediates. The initial oxidation of VLCFA is performed by the *P* *P* *A* *R* *α* inducible acyl-CoA oxidase (AOX1) producing trans-enoyl-CoA, which is sequentially hydrated and dehydrogenated to 3-ketoacyl-CoA by a single bifunctional enzyme, L-enoyl-CoA hydratase/L-3-hydroxyacyl-CoA dehydrogenase (L-BP). The ketoacyl-CoAs are converted to acyl-CoAs and acetyl-CoAs that are acted upon by a group of thioesterases, some induced by PPAR*α*, to acetate and short- or medium- chain fatty acids that are transported to mitochondria for their complete oxidation to CO_2_ and water. Branched-chain fatty acids are metabolized by a noninducible AOX and then further metabolized by the D-bifunctional enzymes (D-enoyl-CoA hydratase/D-3-hydroxyacyl CoA dehydrogenase) to acyl-CoAs, which are cleaved by a specific thiolase known as sterol carrier protein (SCP-X).

The increased expression of the PPAR*α*-dependent peroxisomal *β*-oxidation pathway induced in hepatic steatosis provides an alternative mechanism to remove excessive fatty acids. Impairment of mitochondrial *β*-oxidation is suggested to be an important mechanism of fatty acid induced liver injury. Inhibition of mitochondrial *β*-oxidation can lead to dicarboxylic aciduria [86]; therefore, induction of peroxisomal *β*-oxidation pathway provides an adaptive protective role in reducing intracellular levels of dicarboxylic acids that inhibit the mitochondrial function. The importance of peroxisomal *β*-oxidation in hepatic steatosis is evident in *Aox1*-null mice, which show high levels of VLCFA in serum and serve hepatic steatosis, steatohepatitis, and hepatocellular carcinoma [56]. In lean and fatty (fa/fa) Zucker rats, obese rats showed a 50% reduction in complete mitochondria oxidation and a 3-fold increase in incomplete peroxisomal *β*-oxidation. The increased peroxisomal *β*-oxidation in Zucker obese rats accounted for up to 25% of the total mitochondrial *β*-oxidation by supplying shorter-chain fatty acids to mitochondria for complete oxidation [87]. Therefore, the peroxisomal oxidation of VLCFAs to SCFAs by incomplete *β*-oxidation provides a mechanism by which FAs can be imported into mitochondria directly without activation to a CoA ester and import by CPT1. The peroxisomes have two systems to uptake fatty acids, either as FA-CoA esters by using ATP-binding cassette transports or as free fatty acids. These systems provide a mechanism to prevent lipotoxicity of FFAs in the cytosol. 

However, even though the peroxisomes have the capacity to supply shorter-chain fatty acids to the mitochondria for complete oxidation and an efficient system to remove excessive cytosolic free fatty acids, the *β*-oxidation system is able to generate ROS. The peroxisome-produced H_2_O_2_ accounts for 35% of the total cellular hydrogen peroxide produced and 20% of the total oxygen consumption in hepatocytes [88]. Although peroxisomes have numerous oxidase enzymes that produce H_2_O_2_, they also have several antioxidant enzymes to prevent ROS-mediated cell damage [89]. However, the massive proliferation of peroxisomes in rodents by hypolipidemia drugs and peroxisome proliferator chemicals is a viable reason why rodents develop hepatocarcinogenesis [56]. The central event in rodent hepatocarcinogenesis induced by peroxisome proliferators is the activation of PPAR*α* since *P* *p* *a* *r* *α*-null mice are refractory to peroxisome proliferation and hepatocarcinogenesis when fed the nongenotoxic peroxisome proliferator and PPAR*α* ligand, Wy14,643 [90]. The low levels of PPAR*α* in human liver may be a reason why humans are resistant to the hepatocarcinogenic effects of peroxisome proliferator chemicals despite the beneficial effects of lipid lowering hypolipidemic drugs in the treatment of dyslipidemia.

The apparent beneficial effect of peroxisomal *β*-oxidation in hepatic steatosis is evident in *Aox1-null* mice that develop severe microvesicular steatohepatitis [91, 92]. Similarly, *Ppara*-null mice also develop steatohepatitis [93]. In both mouse models, peroxisomal *β*-oxidation is severely compromised, resulting in increased dicarboxylic acids that uncouple mitochondrial electron transport and inhibits mitochondrial *β*-oxidation pathway. However, in the *Aox1*-null mice, there a massive induction of *CYP4A* genes while *Ppar* *α* *-null* mice do not show induction of *CYP4A* genes. It is unclear about the beneficial role of CYP4A since neither the amount of *ω*-hydroxylated fatty acids nor intracellular level of dicarboxylic acids was determined in these studies [91–93]. However, this paradigm of severe steatohepatitis in *Aox1*-null and *Ppar* *α*-null mice with reduced peroxisomal and mitochondrial *β*-oxidation is questioned in double knock-out (DKO) mice for both genes, which develop only mild steatohepatitis [94]. These results suggest that the role of CYP4A in promoting the severity of steatosis in livers with defective peroxisomal *β*-oxidation is still unclear because of the lack of *P* *P* *A* *R* *α*-mediated induction of *CYP4A* in DKO mice. 

## 7. Regulation of Drug Metabolizing Cytochrome P450 Enzymes in NAFLD

The induction of cytochrome P450 drug metabolizing enzymes has been implicated as a source of ROS in the perpetuation and progression of steatosis to steatohepatitis. Microsomal oxidation of fatty acids, catalyzed by cytochrome CYP2E1, CYP4A10, CYP4A12, and CYP4A14, which are induced in mouse models of steatosis and steatohepatitis, is a potent source of ROS through uncoupling of their catalytic cycles. The production of ROS by cytochrome P450 catalytic cycle is dependent on the redox potential and spin state of the transition element iron of heme [95]. Cytochrome P450 normally functions as a monooxygenase but can work as an oxidase releasing H_2_O_2_ into endoplasmic environment. The P450 catalytic cycle can produce superoxide and peroxide by uncoupling of the substrate metabolism with electron transport [96]. ROS can also be produced by the P450 catalytic cycle by futile cycling and redox cycling (Fe^+2^/Fe^+3^) with the former producing free radical semiquinones and the latter by a similar redox cycling observed in the mitochondria electron transport chain [97]. 

The ethanol-inducible CYP2E1 is elevated in both rodent experimental models of steatosis and steatohepatitis and human NAFLD patients, and in vivo levels of CYP2E1 activation correlate with the severity of liver damage, suggesting that this P450 is one of the major microsomal contributors of ROS-induced hepatic injury [6, 98]. *CYP2E1* is unique among P450s since it is loosely bound to ER membrane and naturally present in the high spin Fe^+3^ state. Therefore it can produce ROS even in the absence of its potentially toxic substrates [99] while its iron is able to interact directly with molecular oxygen [96]. The induction of *CYP2E1* by many potentially toxic/carcinogenic substrates such as ethanol, benzene, and haloalkanes and their metabolisms promote ROS production [6]. Furthermore, the substrates that are poorly metabolized by CYP2E1 such as fatty acids lead to uncoupling and futile catalytic cycle with increased ROS production. Thus, the P450 catalytic cycle generates significant amounts of reactive superoxide anion, substrate radical, and protonation of peroxy-cytochromes along with a third leaky mechanism requiring two-electron transfer followed by decay of peroxycytochrome P450 with the release of superoxide. The efficiency of electron transfer from NADPH to P450 catalytic cycle is called the degree of coupling, which is less than 50% or lower in eukaryotes [96]. Due to the high uncoupling rates of the P450 catalytic cycle, the rates of NADPH and oxygen consumption are weakly dependent on the presence of a substrate. Therefore, the microsomal P450 monooxygenase system is a significant contributor of ROS formation in hepatocytes. 

*CYP2E1* seems to play a key role in the pathogenesis of alcoholic liver injury because of its induction in chronic alcohol drinkers and ability to produce ROS [100]. Increased CYP2E1 protein levels are observed in animal models of fatty liver diseases and in morbid obese men with NAFLD and patients with NASH [101, 102]. CYP2E1 P450 levels are increased in patients with NASH because fatty acids and ketone bodies including acetone, which increased in diabetes/ketosis [96], are substrates and inducers of CYP2E1 protein. However, even with this apparently close association of CYP2E1 with both NAFLD and NASH and in several animal models of both alcoholic and nonalcoholic steatosis, *Cyp2e1*-null mice still develop diet-induced NASH or alcoholic liver inflammatory disease indicating that *Cyp2e1* deletion neither prevented nor decreased oxidative damage [103–105]. However, these *Cyp2e1*-null mice did show a dramatic increase in the amounts of *CYP4A10* and *CYP4A14* fatty acid omega hydroxylases, and inhibition of *CYP4A14 *P450 prevented oxidative damage in *Cyp2e1*-null mice [104], thus demonstrating an important role of *CYP4A* P450 isozymes in the steatohepatitis induced by a MCD diet. 

The omega fatty acid hydroxylase P450s (*CYP4A*) metabolize a variety of endogenous saturated and unsaturated fatty acids that are sequentially dehydrogenated to their corresponding dicarboxylic acids in the cytosol [106]. Dicarboxylic acids are activated by *dicarboxyl CoA synthetase* (ACSVL1) CoA esters that are chain shortened by peroxisomal *β*-oxidation. Although microsomal *ω*-oxidation of fatty acids and peroxisomal *β*-oxidation are minor pathways for fatty acid oxidation, under fatty acid overload, significant amounts of dicarboxylic acids are formed in patients with NAFLD, obesity, and diabetes. Dicarboxylic acids are highly toxic to mitochondria and therefore efficient disposal is necessary to prevent mitochondrial dysfunction during hepatic steatosis. PPAR*α* agonists and fatty acids elevated during fasting and hepatic steatosis could prevent dicarboxylic acid formation through induction of the genes including *ω*-oxidation CYP4A and peroxisomal *β*-oxidation. 

Although the ethanol-inducible *CYP2E1* and fatty acid metabolizing *CYP4A* P450s may be directly involved in the progression of steatosis to steatohepatitis by production of ROS and initiation of lipotoxicity, other drug metabolizing cytochrome P450 genes are dramatically affected in fatty liver disease and therefore have an important role in identification of appropriate drug treatment modalities to avoid the consequences of adverse drug reactions or drug toxicity. In primary human hepatocytes isolated from liver with macrosteatosis, there is a 60% to 40% reduction in 7-ethoxycoumarin *O*-deethylation (ECOD) and testosterone oxidation with a reduction in *CYP1A2*, *CYP2C9, CYP2E1, *and *CYP3A4* mRNA and their respective proteins to metabolize their specific substrate drugs [107]. A recent study analyzed changes in hepatic cytochrome P450 mRNA, protein and enzymatic activity in human patients with steatosis, steatohepatitis and hepatitis without steatosis, which reflects the beginning of liver fibrosis [108]. These livers therefore represent the progressive stages of NAFLD to NASH and ultimately fibrosis. During NAFLD progression, *CYP2E1*, *CYP2C19,* and *CYP1A2* mRNA and the corresponding P450 protein contents were decreased while those of *CYP2A6*, *CYP2B6* and *CYP2C9* mRNA and the proteins were increased [109]. During disease progression, *CYP2D6*, *CYP3A4* or *CYP2C*8 mRNA levels did not change; however CYP3A4 and CYP2D6 protein levels decreased. The changes in CYP P450 levels correlated with the changes in the enzymatic activities of the different P450s in the progression of NAFLD. The differential expression of P450 in the progression of NAFLD to NASH and/or fibrosis has an important clinical implication in patient treatment to avoid adverse drug reactions and possible drug toxicity. CYP2C9 is the second most abundant P450 expressed in human liver and is responsible for the metabolism of *S*-warfarin, Tamoxifen, fluoxetine, losartan, and the antidiabetic PPAR*γ* agonist rosiglitazone. The increase in *CYP2C9* mRNA, protein, and enzymatic activity during NAFLD progression would suggest that the standard dose of rosiglitazone to treat patients with type II diabetes may be ineffective in managing hyperglycemia in patients with NASH or liver fibrosis. CYP3A4 is the most prominent P450 expressed in the human liver and metabolizes over 50% of all therapeutic drugs prescribed. Therefore, reduced CYP3A4 levels during the progression of NAFLD to fibrosis and liver cirrhosis put these patients at risk for adverse drug reaction and possible drug toxicity [110]. In contrast to the many studies indicating a role of *CYP2E1* in NAFLD and NASH [109], the decrease in *CYP2E1* mRNA and protein from steatosis to steatohepatitis and fibrosis needs to be confirmed independently. However, these data question the role of *CYP2E1* in oxidative damage-induced disease progression and suggest a possibility that other P450 enzymes such as CYP4A isozymes elevated in NAFLD and NASH may be involved in the progression of fatty liver diseases. 

## 8. Microsomal Fatty Acid Oxidation in Hepatic Steatosis

The *ω*-hydroxylation of saturated and unsaturated fatty acids by *CYP4* family members has long been thought to be a minor pathway in the metabolism of fatty acids accounting for 5%–10%. However, its importance is dramatically increased due to their upregulation during fasting, starvation, and in several human diseases where its contribution to fatty acid metabolism increases dramatically to 15%–30%. The close association between microsomal CYP4 *ω*-hydroxylation of MCFAs and peroxisomal *β*-oxidation of LCFAs is evident by the conversion of dicaboxylic acids to succinate, an anaplerotic gluconeogenic precursor, and acetate, which can be used by peripheral tissue like ketone bodies during fasting and starvation through a dramatic induction of *CYP4A* genes. The increased expression of *CYP4A* genes during fasting, starvation, by a high fat diet and in steatohepatitis may be a mechanism to prevent lipotoxicity from FFAs, but at the expense of possibly increased uncoupling of the P450 catalytic cycle, leading to increased ROS production. 

In mammals, six *CYP4* gene subfamilies have been identified: *CYP4A*, *CYP4B*, *CYP4F*, *CYP4V*, *CYP4X*, and *CYP4Z* [111–114]. Three of these subfamily members (i.e., *CYP4A*, *CYP4B*, and *CYP4F*) have been shown to *ω*-hydroxylate saturated, branched, unsaturated fatty acids and the eicosanoids. Members of the *CYP4B *subfamily metabolize SCFAs (C_7_–C_9_), while members of the *CYP4A* subfamily metabolize MCFAs (C_10_–C_16_), and members of the *CYP4F* subfamily metabolize LCFA and VLCFA (C_18_–C_26_) fatty acids. 

Members of the *CYP4A* family are by far the best characterized *ω*-fatty acid hydroxylases in regard to their induction by peroxisome proliferators, PPAR*α*, and regulation by fasting, high fat diet, ethanol consumption, and in diabetes in rodents. The importance of *CYP4A* P450s in the metabolism of MCFAs is evident by their upregulation during starvation, caloric restriction, and in animals fed a high fat diet, which mimics starvation-induced lipolysis and excessive fatty acid transport to the liver. In these situations, there is a dramatic induction of the *CYP4A* genes, which may function to not only prevent lipid toxicity but also provide consumable nutrients for peripheral tissue during starvation. MCFAs in hepatocytes are transported into the peroxisomes as FFAs or as dicarboxylic acids after *CYP4A* *ω*-hydroxylation and esterified by *peroxisomal acyl-CoA synthetase* ACSVL1 (FATP2) and ACSVL5 (FATP4) [26]. MCFA acyl-CoAs undergo 2 to 3 rounds of peroxisomal *β*-oxidation, producing succinyl-CoA and acetyl-CoA [115, 116]. These products are converted by several peroxisomal acyl-CoA thioesterases (ACOT), which can catalyze the hydrolysis of CoA esters of different chain-length fatty acids including succinate. Succinate can be directly used as an anaplerotic intermediate for gluconeogensis while released acetate can be taken up and oxidized by extra-hepatic tissues in the same way as ketone bodies for energy production. During starvation or administration of hypolipidemic drugs that activate PPAR*α*, there is a rapid proliferation of peroxisomes in rodents but not humans. In humans, the *CYP4A11* and *CYPF2* genes are not induced by peroxisome proliferators (PP), and therefore the absence of peroxisome proliferation may be due to decreased levels of *ω*HEET, which is a high affinity ligand in the PPAR*α* activation. In humans, the hypolipidemic effect of peroxisomal proliferators is not mediated through PPAR*α* activation but through the suppression of HNF4*α* by PPs-CoAs [55]. HNF4*α* controls genes involved in the production of lipoproteins [55]. Thus, the activation of PPAR*α* in rodents by *ω*HEET and the suppression of HNF4*α* in humans by PP-CoAs may explain the absence of peroxisome proliferation in humans and why humans in contrast to rodents are resistant to the hepatocarcinogenic effects of hypolipidemic drugs. 

In contrast to the *CYP4A* members, *CYP4F* P450s isozymes *ω*-hydroxylate a variety of LCFAs and VLCFAs, unsaturated and branched-chain fatty acids,andvitamins with long alkyl side chains, the physiologically important leukotrienes (LT), prostaglandins (PG), and hydroxyeicosatetraenoic acids (HETE) [117–121]. The human *CYP4F* P450s metabolize and inactivate the proinflammatory leukotriene B_4_ (LTB_4_) [118], with the myeloid-expressed CYP4F3A having a twofold greater affinity for LTB_4_ than CYP4F2 expressed in liver, kidney, and skin, but not in myeloid cells. However, *CYP4F3B* splice variant of the *CYP4F3* gene expressed in liver and has a similar affinity as CYP4F2 for LTB_4_ [122]. Both CYP4F3B and CYP4F2 P450 can metabolize arachidonic acid to 20-HETE while CYP4F3A has little activity towards arachidonic acid. Besides *ω*-hydroxylating other proinflammatory eicosanoids such as 5-HETE, 12-HETE, and 8-HETE, *CYP4F* P450s can metabolize the anti-inflammatory lipoxins, LXA_4_, and LXB_4_. The ability of CYP4F3 and CYP4F2 to *ω*-hydroxylate both pro- and anti-inflammatory leukotrienes indicates that they may function both in the activation and resolution phases of the inflammatory response [106, 113]. 

Similar to the MCFAs and SCFAs, omega-hydroxylated LCFAs and VLCFAs are converted to their corresponding dicarboxylic acids by the sequential action of cytosolic *alcohol *and *aldehyde dehydrogenases*. The roles of different chain length *ω*-hydroxylated fatty acids in lipid metabolism are indicated by increased *ω*-hydroxylation of MCFAs by *CYP4A* P450s in rodents during fasting, by peroxisome proliferators, and in hepatic steatosis while decreased *CYP4F* genes expression by peroxisome proliferators and during starvation results in reduced *ω*-hydroxylation of LCFAs and VLCFAs. Unlike the *ω*-hydroxylated MCFAs, which are *β*-oxidized to succinyl-CoA and acetate, the omega-hydroxylation of LCFAs would produce only SCFAs and acetate. Excessive acetate in the hepatocyte cytosol [123] can be used for the synthesis of cholesterol and fatty acids with malonyl-CoA inhibiting the mitochondrial CPT1 fatty acid uptake and therefore blocking mitochondrial *β*-oxidation. Dicarboxylic acids are almost elusively metabolized by the peroxisomal *β*-oxidation system since the Km value for dicarboxylic acids by the mitochondrial system is 15–40-fold higher than that of the peroxisomes [124]. Furthermore, branched and long chain saturated and unsaturated fatty acids are preferentially metabolized by the peroxisomes. The transportation of fatty acids into the peroxisomes is different for MCFAs, LCFAs, and VLCFAs. MCFAs are transported as free acids, which are esterified in peroxisomes to their corresponding CoA derivatives by FATP2 and FATP4, while LCFAs and VLCFAs are transported by ABC transporters as CoA derivatives [125]. In addition, *β*-oxidation in the peroxisomes does not completely oxidizes fatty acid substrates but produces shorter-chain fatty acids which are transported from the peroxisomes to mitochondria for either complete oxidation by the mitochondrial *β*-oxidation or usage for synthesis of other fatty acids, as seen in the conversion of C24:6n-3 to docosahexaenoic acid (C22:6n-3). 

Omega-hydroxylated fatty acids and eicosanoids have several metabolic fates dependent upon the cell type and *CYP4* genes expressed. In the liver, *ω*-hydroxylated fatty acids can be metabolized and used for energy production, lipogenesis, the synthesis of structural lipids, and production of fatty acids that function as agonists in the regulation of hormone nuclear receptors (HNRs) (Figure 1). The diverse array of fatty acids omega-hydroxylated by members of the *CYP4* family and their functional roles in metabolism, cell signaling, inflammation, and lipid structure suggest that CYP4 members play an important role in human diseases [106]. The importance of *ω*-hydroxylated fatty acids and eicosanoids in human diseases is evident by the role 20-HETE has in hypertension and vascular disease and its dramatic appearance in patients with end stage liver diseases, and the possible role of *ω*-hydroxylated fatty acids in lipid metabolism in fatty liver diseases [113, 126–128].

## 9. Role of CYP4 Isozymes in the Initiation and Progression of NAFLD to NASH

NAFLD encompasses a broad disease spectrum ranging from simple triglyceride accumulation in hepatocytes (hepatic steatosis) to hepatic steatosis with inflammation (steatohepatitis) that can progress to fibrosis and cirrhosis [1]. A two-hit hypothesis has been proposed to explain the progression of NAFLD as hepatic triglycerides being the first hit, while increased oxidative stress, as the second hit, promoting liver inflammation, cell death, and fibrosis in NASH [127, 129]. Both obesity and insulin resistance are strongly associated with NAFLD, resulting in the hydrolysis of adipose triglycerides by hormone sensitive lipases, leading to elevated plasma and hepatic levels of FFAs. In the liver FFAs can be either metabolized by the mitochondrial *β*-oxidation system for energy production or esterified to triglycerides and incorporated into VLDL particles with cholesterol esters and phospholipids for transport and use by peripheral tissues. Through the use of mouse models of fatty liver disease and the generation of knockouts of key regulator enzymes involved in lipid and glucose metabolism, we are beginning to understand the key molecular targets responsible for increased triglyceride accumulation in hepatocytes and how fatty acids induce oxidative stress and the progression of steatosis to steatohepatitis. 

Key regulatory enzymes control the ability of the liver to provide nutrients to peripheral tissues through gluconeogenesis, ketogenesis, VLDL secretion and possibly acetate. Interestingly, key regulatory enzymes are differentially affected in liver insulin resistance with the major pathway in lipogenesis being activated while the hepatic gluconeogenic pathway is active under insulin resistance. Both a*cetyl-CoA carboxylases* (ACC1 and ACC2) and *stearoyl-CoA desaturase* (SCD-1) are activated in the insulin resistant liver. ACC1 is responsible for converting acetyl-CoA to malonyl-CoA, which functions as both a precursor of cholesterol and fatty acid synthesis and also a potent inhibitor of mitochondrial CPT1 necessary for the transport of fatty acids to the mitochondria for *β*-oxidation while SCD-1 functions to desaturate stearic acid (C_18:0_) to oleic acid (C_18:1_) necessary for synthesis of triglycerides. These genes are activated through insulin-mediated activation of two transcription factors (e.g., SREBP-1c and ChREBP). During hepatic steatosis in both humans and mouse models, there is an excessive accumulation of oleic acid either from increased de novo fatty acid synthesis or conversion of imported fatty acids from the adipose tissues. Elevated hepatic glucose production in the presence of hyperinsulinemia is a hallmark of insulin resistance in the liver even with the increased expression of the liver specific *pyruvate kinase* (L-PK), a key *regulatory* enzyme in converting phosphoenolpyruvate to pyruvate during glycolysis. Increase *L-PK* gene expression is mediated by glucose activation of the ChREBP transcription factor. ChREBP has also been shown to increase the expression of many of the fatty acid synthesis enzymes as well as SCD-1, thereby facilitating the conversion of glucose to fatty acids. It is presently unknown why hyperinsulinemia activates the lipogenic pathway, but fails to prevent the inactivation of a gluconeogenic pathway and hyperglycemia observed in insulin resistant diabetes [127, 129, 130]. 

The activation of both anabolic and catabolic pathways during insulin resistance in the liver suggests that different fasting and feeding signals are simultaneously controlling the metabolic responses of the liver. What these signals are and how they function either directly to activate key regulator enzymes or as agonists or antagonists to activate key transcription factors that control the expression of the genes involved in fatty acid catabolism (PPAR*α*, HNF4*α*) or fatty acid synthesis (SREBP-1c, ChREBP, PPAR*γ*) remain to be determined.

The increase in peroxisomal *β*-oxidation during steatosis exerts a beneficial effect in NAFLD by metabolizing excessive fatty acids to shorter-chain fatty acids that can be directly transported and completely oxidized by the mitochondrial *β*-oxidation system. In addition, the incomplete peroxisomal *β*-oxidation of fatty acids can supply the anaplerotic mitochondrial intermediate, succinate, necessary for gluconeogenesis, while acetate from acetyl-CoA can be used for the anabolic synthesis of cholesterol and fatty acid as seen in NAFLD [131–133]. The peroxisomes, unlike mitochondria, metabolize long chain fatty acids, exclusively metabolize branched-chain fatty acid, and preferentially metabolize dicarboxylic acids, which are produced by the *ω*-hydroxylation of fatty acids by members of the *CYP4* gene family. The increased expression of the *CYP4A* omega-hydroxylases during steatohepatitis and their induction in animals fed with a high-fat diet suggest that they may play a pivotal role in lipotoxicity [134], and may be responsible for the induction of oxidative stress as well as progression to steatohepatitis. A dramatic induction of both the mouse *CYP4A10* and *CYP4A14* genes is seen in *Cyp2e1*-null mice and likely accounts for the increased ROS that induce lipid peroxidation [103, 104]. The increased production of dicarboxylic acids during steatosis by *CYP4A* members can impair mitochondrial function by dissipation of the mitochondria proton gradient and uncoupling of oxidative phosphorylation. In addition, the uncoupling of the P450 catalytic cycle has been known to be a major source of ROS, which led to the identification of the ethanol-inducible CYP2E1 P450 as a major source of ROS-mediated microsomal lipid peroxidation [96]. CYP2E1 can metabolize fatty acids at the *ω*-1 position, and CYP4A, which normally *ω*-hydroxylates lauric acid and hydroxylates longer chain fatty acids at both the *ω*- and *ω*-1 positions. It is not known whether different chain length fatty acids assist in the uncoupling of the CYP2E1 and CYP4A catalytic cycles or whether cytochrome b_5_, which increases P450 catalytic activity and prevents uncoupling, can reduce ROS formation in fatty liver disease [135]. Both cytochrome b_5_ reductase and cytochrome b_5_ are also used in the desaturation of stearic and palmitic acids by SCD-1. It is unknown whether increased conversion of stearic acid to oleic acid observed in NAFLD increases uncoupling of the P450 catalytic cycle, resulting in increased ROS formation by SCD-1 sequestering cytochrome b_5_. Both cytochrome b_5_ and cytochrome b_5_ reductase have been identified as susceptibility genes in obesity [136]. While the induction of *CYP4A* genes during fasting provides both gluconeogenic precursors and acetate to supply the needs for peripheral tissues, their induction during steatosis may increase hyperglycemia, shuttle acetate for synthesis of fatty acids and cholesterol, and increase ROS formation by uncoupling of the P450 catalytic cycle. 

Similar to the role of *CYP4A* genes in possibly initiating hepatocyte cell injury and steatohepatitis, *CYP4F* genes may also play a functional role in hepatic lipid accumulation and in the recruitment of inflammatory cells during progression from steatosis to steatohepatitis. Unlike *CYP4A* genes that are induced by fasting, hypolipidemic drugs, and peroxisome proliferators through PPAR*α* activation, the *CYP4F* genes were reported to be repressed during fasting and by peroxisome proliferators possibly by PP-CoA or PUFA-CoA mediated inhibition of HNF4*α* [137]. Furthermore, unlike the *CYP4A* genes that are induced in fatty liver, our preliminary data suggest that the mouse *CYP4F* genes may be repressed in mice fed a high fat diet and in the leptin-deficient ob/ob mice as a model of fatty liver disease (unpublished results). The recent report of lipid accumulation in primitive liver cells (oenocytes) of Drosophila that have a mutation in the *stearic* *ω* *-hydroxylase CYP4g1* gene [138] suggests that *CYP4F *genes may play an important role in maintaining lipid homeostasis in the liver. Drosophila homozygous mutant for *CYP4g1* manifests a two-fold increase in the oleic acid  :  stearic acid ratio (C18  :  1/C18  :  0) with a notable imbalance in the fatty acid desaturation found in the TAG fraction but not in the phospholipid fraction. This suggests that CYP4g1 is important in the metabolic storage of fatty acids and its expression would decrease oleic acid synthesis and storage of fatty acids in TAGs. The human CYP4F2 efficiently *ω*-hydroxylates stearic acid and therefore may serve the same function as CYP4g1 in competing with SCD-1 in the metabolism of stearic acid [117]. It is currently unknown whether the human *CYP4F2* gene is repressed by fatty acids in patients with NAFLD. However, one study suggested that the human *CYP4A11* mRNA was reduced in obese individuals [126]. In contrast, the *CYP4F2* gene has been shown to be downregulated by peroxisome proliferators [139], induced by retinoic acids [140], and its expression increased by lovastatin-mediated activation of SREBP-2 [141]. 

Therefore, the activation of the *CYP4F2* gene in fatty liver diseases may decrease the formation of oleic acid and storage of TAG in the liver, and also play a vital role in preventing recruitment of immune cells to the liver during steatohepatitis by metabolism of the proinflammatory leukotrienes. Even though there are numerous studies showing that fatty acids induce hepatocytes to produce proinflammatory cytokines and chemokines (IL-8) which attract neutrophils to the liver, the function of leukotrienes in attracting immune cells to the liver during steatohepatitis has not been explored. Whether LTB_4_ or LTC_4_ increases production of the potent neutrophil chemokine IL-8 during hepatic steatosis remains to be determined. The genetic association of *CYP4F2* and the neutrophil-specific *CYP4F3A* gene in Celiac disease establishes a connection between the innate immune response of neutrophil recruitment to the established Th1 adaptive immune response in disease patients [142]. Furthermore, Celiac disease has been associated with several inflammatory diseases of the liver including primary biliary cirrhosis, primary sclerotizing cholangitis, autoimmune hepatitis, hemochromatosis, and fatty liver diseases [143]. 

It will be of significant importance to understand how the *CYP4F *genes are regulated by fatty acids and in animal models of fatty liver disease. Whether induction of CYP4F long chain fatty acid *ω*-hydroxylases can prevent steatosis by inhibiting SCD-1 activity and reduce the hepatic levels of proinflammatory leukotrienes in steatohepatitis needs further study.

## 10. Genetic Regulation of CYP4A and CYP4F Genes and Their Roles in Lipid Metabolism during Hepatic Steatosis

The differential regulation of the *CYP4A* and *CYP4F* genes by peroxisome proliferators, during fasting and by high fat diets indicates that these P450s may have distinct roles in lipid metabolism during the fasting and feeding cycles, and that different nuclear transcription factors regulate the expression of these genes (Figure 1). It is well established that the rodent *CYP4A* genes are induced by or during hepatic steatosis and steatohepatitis [104, 134, 144, 145]. However, their functional roles in initiation and progression of NAFLD to NASH are relatively unknown. The importance of induction of *CYP4A *genes in hepatic steatosis and steatohepatitis was revealed in *Cyp2e1*-null mice that developed severe steatohepatitis with a pronounced increase in CYP4A10 and CYP4A14 associated with increased accumulation of lipid peroxides. The knockout mice deficient of the peroxisome straight chain acyl-CoA oxidase (*Aox-*null) fed a normal diet develop severe steatohepatitis with a massive increase in CYP4A protein expression. In contrast, *P* *p* *a* *r* *α*-null and *Aox*-null double-knockout mice develop only mild to moderate steatohepatitis with much less lipid peroxide accumulation because a CYP4A protein, which produces ROS and lipid peroxidation, was not induced. It is known that PPAR*α* agonists exhibit a beneficial effect on preventing steatohepatitis by increasing fatty acids *β*-oxidation and indirectly by inhibiting SREBP-1c, thus protecting against obesity-induced hepatic inflammation [146, 147]. To resolve this issue, Leclercq and colleagues fed wild type and *P* *p* *a* *r* *α*-null mice with a MCD diet to induce steatohepatitis and the specific PPAR*α* agonist, Wy14,643 [145]. MCD-diet fed *P* *p* *a* *r* *α*-null mice, which are very sensitive to oxidative stress [148], develop severe steatohepatitis in the absence of *CYP4A* induction and, Wy14,643 decreased the degree of steatohepatitis in these mice. These data suggest that *CYP4A* induction was not necessary for promoting steatohepatitis or the cause of increased microsomal lipid peroxides in *P* *p* *a* *r* *α*-null mice fed a MCD diet [145]. It is difficult to reconcile the effect of the PPAR*α* agonist in the prevention of steatohepatitis in *P* *p* *a* *r* *α*-null mice since none of the mitochondrial, peroxisomal *β*-oxidation, and microsomal *ω*-oxidation pathways was induced by Wy14,643 in these mice. These results indicate that inflammatory cells might be responsible for increased lipid peroxidation and steatohepatitis in *P* *p* *a* *r* *α*-null mice. These data further suggests that Wy14,643 may have a *P* *P* *A* *R* *α* independent effect on the amelioration of steatohepatitis. In fact, another *P* *P* *A* *R* *α* agonist bezafibrate at clinically relevant doses decreased serum and liver triglycerides through down-regulation of SREBP-1c, revealing a clue for *P* *P* *A* *R* *α*-independent mechanism in the suppression of de novo lipogenesis [146]. In contrast, wild-type mice fed the MCD diet and Wy14,643 do not develop steatohepatitis, although both the rates of peroxisomal and mitochondrial *β*-oxidation are increased with a 20~50-fold-fold upregulation in *CYP4A10* and *CYP4A14* gene expression. Thus, these results further question the role of ROS generation from either mitochondrial *β*-oxidation or microsomal *ω*-oxidation in the development of steatohepatitis. To resolve this issue and to further define the role of *CYP4A* genes in production of ROS during hepatic steatosis, mouse models overexpressing *CYP4A10 *and *CYP4A14 *will be needed to determine if excessive fatty acid induces uncoupling of the P450 catalytic cycle and generation of ROS during the progression of NAFLD to NASH. However, most PPAR*α* agonists activate the expression of the *CYP4A* genes, suggesting an interesting paradox whether *CYP4A* gene induction is beneficial or harmful in promoting steatohepatitis. A recent study suggests that *CYP4A14* overexpression in hyperoxia increases resistance to oxidative stress [149]. In contrast to the extensive data on the role of rodent *CYP4A* gene in animal models of steatosis and steatohepatitis, little is known about how the human *CYP4A11* gene is controlled by PPAR*α* agonists and its functional role in NAFLD or NASH. The modest 2-fold induction of the *CYP4A11* gene by peroxisome proliferators in primary human hepatocytes compared with the 30–70 fold induction of the mouse *CYP4A* genes indicates a species difference between rodents and humans with respect to regulation of the *CYP4A *genes by peroxisome proliferators [150]. In addition, the 60–700 fold increase in mouse *CYP4A *mRNA during fasting [144] and 2–8 fold decrease in *CYP4F* mRNA further indicate the differential regulation of these distinct genes. In humans with obesity, *CYP4A11* mRNA decreased by 50% while in NAFLD patients while *CYP4A11* mRNA increases 4-fold [126], suggesting a differential regulation in liver disease progression.

LCFAs are endogenous ligands in the activation of NHRs, PPAR*α* and HNF4*α*. LCFAs and LCFA-CoAs are significant NHR ligands as shown by their presence in the nucleus, their high affinity binding (Kd values ~ nM ranges), their ability to induce conformational changes in NHRs, and their ability to induce coregulator recruitment to nuclear receptors [55]. Support for LCFA-CoAs in the hyperactivation of *P* *P* *A* *R* *α* was evident in *Aox-*null mice with accumulated VLCFAs and VLCFA-CoAs, and the observation that the thio-esterification inhibitor, 2-bromopalmitate inhibits bezafibrate induced peroxisome proliferation in rodents. In humans, the importance of VLCFA-CoAs in PPAR*α* activation was evident in adrenoleukodystrophy where there is accumulation of VLCFA in the cytosol, but no peroxisome formation of VLCFA-CoA and no hyperactivation of PPAR*α* [120]. Serum fatty acids increase dramatically from the normal physiological range of 200 uM to 1 mM under fasting and up to 8 mM in Refsum's disease, adrenoleukodystrophy, Zellweger's syndrome, and fatty liver diseases, diabetes, and inflammation. This suggests an important link between peroxisome fatty acid metabolism and conversion of VLCFAs to VLCFA-CoAs in the activation of PPAR*α* and control of *CYP4* gene expression. PPAR*α* has a high affinity for polyunsaturated LCFAs, LCFA-CoAs and VLCFA-CoAs, but not saturated LCFAs or VLCFAs while HNF4*α* has a high affinity for saturated LCFAs and VLCFA acyl-CoAs but not polyunsaturated acyl-CoAs (Figure 1). These facts indicate that fatty acid CoA chain length and degree of unsaturation determine whether HNF4*α* or PPAR*α* will be activated [48]. The mechanism of LCFA uptake and importation into the nucleus has recently been shown to be mediated by L-FABP, which binds polyunsaturated LCFAs with a greater affinity than saturated LCFAs and associates with PPAR*α*, while ACBP preferentially binds saturated LCFAs and associates with HNF4*α* [55]. These studies indicate that ACBP selectively cooperates with HNF4*α* while L-FABP selectively cooperates with PPAR*α*, which is believed to elicit downstream alteration in coactivator and corepressor association with NHRs. Thus, the binding of saturated LCFA-CoAs to HNF4*α* would increase HNF4*α* activity and inhibit PPAR*α* transactivation while polyunsaturated LCFA-CoAs would decrease HNF4*α* activation and increase L-FABP PPAR*α* transactivation. Since PPAR*α* and HNF4*α* regulate gene transcription through similar promiscuous DR1 sequences, and compete for the same coactivators and corepressors, the specificity of receptor activation may be determined by either saturated or polyunsaturated fatty acid ligand while the cross-talk between these transcription factors would be determined by the FABP/ACBP mediated coregulator recruitment and repression of the cognate receptor. For instance, the differential regulation of the *CYP4A* and *CYP4F* genes may be determined by cross-talk between PPAR*α* and HNF*α* through the type of fatty acid ligand, method of nuclear import, and receptor activation by L-FABP or ACBP. This scenario is highly likely in the regulation of *CYP4A* and *CYP4F* genes since peroxisome proliferators (PP) activate PPAR*α* while PP-CoAs inhibits HNF4*α* transactivation. It is also possible that MCFAs and VLCFAs metabolized by the *CYP4A* and *CYP4F* may produce fatty acid metabolites that reciprocally regulate the expression of the *CYP4A* and *CYP4F* genes (Figure 1). The induction of *CYP4A* genes by a high fat diet leads to increased production of dicarboxylic acids that are potent inhibitors of HNF4*α* transactivation, which may contribute to the suppression of the *CYP4F* genes during steatosis.

## 11. Conclusion and Discussion

The concentrations of FFAs increase either in the blood plasma through a high-fat diet and release by adipocytes or in the liver as a consequence of lipolysis or de novo fatty acid synthesis. FFAs travel through the body mainly bound to albumin and intracelluarly bound to fatty acid transport proteins (FABP, FATP), which regulate their intracellular fate. Evidence suggests that FFAs induce insulin resistance by raising intracellular lipid metabolites, which activate protein kinase C that inhibits nuclear factor kappa *β* kinase and activates the inflammatory pathway. Activation JNK and/or p38 kinase especially by saturated FFAs and increased ROS [151, 152] leads to serine phosphorylation and inhibition of insulin receptor substrates IRS-1 and IRS-2, resulting in decreased insulin signaling (i.e., decreased Tyrosine phosphorylation) and increased hepatic gluconeogenesis during insulin resistance. The elevation of plasma FFAs in obesity, fatty liver diseases and insulin resistance are predictors of type 2 diabetes, and therefore understanding the mechanisms to decrease hepatocyte intracellular levels of FFAs will offer opportunities to prevent hepatic lipotoxicity and treat not only fatty liver diseases, but also metabolic syndrome associated with obesity and diabetes. The importance of understanding the mechanism by which PPARs can be used to treat hepatic steatosis is apparent considering that PPAR*α*, although downregulated in fatty liver diseases, is a viable target to increase the mitochondrial, peroxisomal *β*-oxidation, and microsomal *ω*-oxidation of FFAs to prevent lipotoxicity despite production of some ROS by the latter two processes. In hepatic steatosis and NAFLD, hepatic PPAR*γ* levels increase; this compensates for the decreased PPAR*α* and presents another important target to prevent excessive intracellular FFAs by increasing de novo lipogenesis and thus preventing the lipotoxicity from FFAs. It will be of importance to determine if PPAR*γ* is able to be activated by selective fatty acids through interaction with L-FABP or ACBP. Furthermore, recent evidence has suggested that PPAR*δ* is a true sensor of plasma FFA levels by interacting with and stimulating the expression of lpin2 and St3gal5 genes after fasting [153]. Whether selective activation of PPAR*δ* can activate the genes involved in either the disposal or lipogenesis to prevent FFA-mediated lipotoxicity will be important in understanding the pathogenesis of NAFLD and NASH. Although, steatosis is the first step or hit in the progression of NAFLD to NASH, the source of ROS in the second step has not been clearly defined and will require investigations. These may include simultaneous measurement of the source of ROS generated by mitochondria, peroxisome, microsome, and inflammatory cells during the progression of steatosis to steatohepatitis. Finally, based on the similar steps of disease progress between NAFLD and alcoholic fatty liver diseases (AFLD) [7, 152], the similar mechanisms and relevant problems can be also applied to understanding of the pathogenesis mechanisms of AFLD. 

## Figures and Tables

**Table 1:** Nomenclature and properties of fatty acid transport proteins.

Gene id	Nomenclature	Tissue	Regulation	Substrate, ligand, or binding protein	Subcellular location	Function
SLC27A1	FATP1-ACSVL4	Heart, adipose, muscle, brain	PPAR*γ*	C16:0, C18:1, C24:0	Mitochondria	*β*-oxidation
					Plasma	TAG synthesis
					membrane	
SLC27A2	FATP2-ACSVL1	Liver, kidney	PPAR*α*, PPAR*γ*	C16:0, C24:0	Endoplasmic	TAG synthesis
				Phytanic acid,	reticulum	*β*-oxidation
				pristanic acid,	Peroxisome	
SLC27A3	FATP3-ACSVL3	Kidney, ovary, lung, brain, adrenal, testis		C16 :0, C18:1, C24:0	Cytosolic vesicles	unknown
SLC27A4	FATP4-ACSVL5	Liver, kidney, heart, adipose, skin, muscle, small intestine	PPAR*γ*, SREBP1c	C16:0, C24:0	Endoplasmic	TAG synthesis
					reticulum	*β*-oxidation
					Peroxisome	
SLC27A5	FATP5-ACSVL6	Liver		Cholate, THCA	Endoplasmic	Bile acid conjugation
				Chenodeoxycholate	reticulum	Bile acid synthesis
				Lithocholate, C24:0	Peroxisome	
				Deoxycholate		
SLC27A6	FATP6-ACSVL2	Heart, placenta		C18:1, C20:4, C24:0	Plasma membrane	unknown
FABP1	L-FABP	Liver, Intestine	PPAR*α*, HNF4*α*	Acyl-CoA, PPAR*α*, *γ*	Cytosol, nucleus	
FABP2	I-FABP	Intestine		Acyl-CoA	Cytosol	TAG synthesis
FABP3	H-FABP	Heart, kidney	c/EBP*α*, SREBP1	Acyl-CoA, PPAR*α*	Cytosol	*β*-oxidation
		muscle, thymus	AP-1			
FABP4	A-FABP	Heart, adipose,	cJun, PPAR*γ*	Acyl-CoA, PPAR*γ*	Cytosol	Chylomicron
		Epidermis, nerve				assembly
FABP5	E-FABP	Eye, adipose, testis	PPAR*δ*	Acyl-CoA, PPAR*δ*	Cytosol	lipogenesis
FABP6	Il-FABP	Ileum		Acyl-CoA, FXR*α*	Cytosol	
FABP7	B-FABP	Liver, brain	POU	Acyl-CoA		
FABP8	N-FABP	Myelin		Acyl-CoA	Cytosol	Vesicle assembly
FABP9	T-FABP	Testis		Acyl-CoA	Cytosol	
FABP12	R-FABP	Retina,testis		Acyl-CoA	Cytosol	
ACBP	L-ACBP	Liver, multiple tissues	PPAR*α*, c/EBP*α*	C14:0–C22:0 acyl-CoA esters, HNF4*α*	Cytosol	Glycerolipid, cholesterol synthesis
			SREBP1c, Sp1			
			PPAR*γ*			
ACBP	T-ACBP	Testis, adrenal		C14:0–C22:0 acyl-CoA esters	Cytosol, endoplasmic reticulum	
ACBP	B-ACBP	Brain		C14:0–C22:0 acyl-CoA esters	Cytosol	

Characteristics of fatty acid transport protein (FATP-ACSVL), fatty acid binding protein (FABP), and acyl-CoA binding protein (ACBP). This table summarizes tissue specific expression, regulation by transcription factors, substrate, ligand binding, and interaction with nuclear receptors and putative function in the metabolism of fatty binding proteins.

## Abbreviations

PUFA: Polyunsaturated fatty acid
MUFA: Monounsaturated fatty acid
SFA: Saturated fatty acid
MCFA: Medium chain fatty acid
LCFA: Long chain fatty acid
VLCFA: Very long chain fatty acid
NAFLD: Nonalcoholic fatty liver disease
NASH: Nonalcoholic steatohepatitis
FFA: Free fatty acid
PPAR*α*: Peroxisome proliferator activated receptor *α*
SREBP: Sterol regulatory binding proteins (SREBP-1 and SREBP-2)
LXR*α*: Liver X receptor *α*
ChREBP: Carbohydrate response element binding protein
ACBP: Acyl CoA binding protein
MTP: Microsomal triglyceride transfer protein
FABP: Fatty acid binding protein
FATP: Fatty acid transport protein
HNF4*α*: Hepatocyte nuclear factor 4
DNL: De novo lipogenesis
NEFA: Nonesterified fatty acids
ROS: Reactive oxygen species
LTB_4_: Leukotriene B_4_
20-HETE: 20-hydroxyeicosatetrenoic acid
CYP: Cytochrome P450
LDL: Low density lipoprotein
HDL: High density lipoprotein
VLCL: Very low density lipoprotein
SCD-1: Stearoyl CoA-desaturase
JNK: c-Jun N-terminal protein kinase
IRS: Insulin receptor substrate
DAG: Diacylglycerol
PEPCK: Phosphoenolcarboxylase kinase
G6Pase: Glucose 6-phosphatase
UCP: Uncoupling protein
L-BP: L-bifunctional protein
HEET: Hydroxyepoxyeicosatetraenoic acid
PP-CoA: Peroxisome proliferator-CoA
CPT-1: Carnithine palmitoyltransferase
DHA: Docosahexaenoic acid
L-PK: Liver pyruvate kinase
ACC: Acyl-CoA carboxylase
IL-8: Interleukin 8
TAG: Triacylglycerol
MCD: Methionine-choline deficient diet
Lpin2: Phosphatide phosphohydrolase
st3gal5: Monosialoganglioside synthase (GM3 synthase).
